# *BMC Ecology* reviewer acknowledgement 2015

**DOI:** 10.1186/s12898-016-0060-5

**Published:** 2016-02-11

**Authors:** Julia Simundza

**Affiliations:** BioMed Central, 233 Spring Street, New York, NY 10013-1578 USA

## Abstract

The editors of BMC Ecology would like to thank all our reviewers who have contributed to the journal in Volume 15 (2015).

The editors of *BMC Ecology* would like to thank all our reviewers who have contributed to the journal in Volume 15 (2015).

**Jean François Agnèse**

France

**Elizabeth Archie**

USA

**Maxwell Asante**

Ghana

**Norbert Becker**

Germany

**Michaela Blyton**

Australia

**Evelyne Buffan-Dubau**

France

**Lyubov Burlakova**

USA

**Michael Butler**

USA

**Brenda Casper**

USA

**Barbara Caspers**

Germany

**Sarah Castle**

USA

**Mathieu Chevalier**

France

**Dong-Hwan Choe**

USA

**Tatiana Cornelissen**

Brazil

**Rosa Cuevas**

Philippines

**Pieter De Frenne**

Belgium

**Carsten Dormann**

Germany

**Victoria Dreitz**

USA

**Simon Ducatez**

Australia

**Karl Duffy**

South Africa

**Leila El Masri**

Austria

**Ahmed El-Gabbas**

Germany

**Leif Engqvist**

Switzerland

**Karl Evans**

UK

**Marie-Anne Felix**

France

**Pablo Ferreras**

Spain

**Magne Friberg**

Sweden

**Matthias Galipaud**

Germany

**Gabriel Garcia-Peña**

France

**Alexander Georgiev**

USA

**Joel Gibson**

Canada

**José Fernando González-Maya**

Mexico

**Jérôme Guélat**

Switzerland

**Rebecca Hale**

USA

**Peter Hamback**

Sweden

**Simon Harvey**

UK

**Kenny Helsen**

Belgium

**Nina Hobbhahn**

South Africa

**Elizabeth Jeffers**

UK

**Jaime Jiménez**

USA

**Eelke Jongejans**

Netherlands

**David Joubert**

Namibia

**Maiko Kagami**

Japan

**Hannele Kauranen**

Finland

**Yuta Kawarasaki**

USA

**Danny Kessler**

Germany

**Erik Kleyheeg**

Netherlands

**Sonja Knapp**

Germany

**Mirjam Knoernschild**

Germany

**Jaroslaw Kobak**

Poland

**Massimo Labra**

Italy

**Paméla Lagrange**

Canada

**Pietro Landi**

South Africa

**Kevin Langergraber**

Germany

**Guillaume Latombe**

Australia

**Franck Lejzerowicz**

Switzerland

**O. Thomas Lorenz**

USA

**Keping Ma**

China

**Stephen Martin**

UK

**Stefan Meldau**

Germany

**Moritz Mittelbach**

Germany

**Liesje Mommer**

Netherlands

**Jesse Morris**

USA

**Teruyoshi Nagamitsu**

Japan

**Shinichi Nakagawa**

New Zealand

**Massimo Nepi**

Italy

**Casper Nyamukondiwa**

Botswana

**Clint Oakley**

New Zealand

**Örjan Östman**

Sweden

**Raghuveer Parthasarathy**

USA

**Arunava Pattanayak**

India

**Robert Paxton**

Germany

**Rachel Penczykowski**

Finland

**Vlada Peneva**

Bulgaria

**Christophe Piscard**

France

**Aimara Planillo**

Spain

**Janos Podani**

Hungary

**Erik Poelman**

Netherlands

**Jérôme Prunier**

France

**Martin Pullan**

UK

**Petr Pysek**

Czech Republic

**Guangyuan Rao**

China

**Rodney Richardson**

USA

**Alex Richter–Boix**

Sweden

**Carlo Ricotta**

Italy

**Gustavo Romero**

Brazil

**Jenna Ross**

South Africa

**Omar Rota-Stabelli**

Italy

**Carlos Rouco**

New Zealand

**Luis Martin San José**

Switzerland

**Boris Valentijn Schmid**

Norway

**Bob Scholes**

South Africa

**André P. Seale**

USA

**Eyal Seroussi**

Israel

**Colleen Seymour**

Somalia

**Ahmed Siddig**

USA

**Stefan Siebert**

South Africa

**Emily Solly**

Switzerland

**Shucun Sun**

China

**Riin Tamme**

Estonia

**Masaatsu Tanaka**

Japan

**Michael Taylor**

Austria

**Mike Taylor**

New Zealand

**Pablo Tedesco**

France

**Reid Tingley**

Australia

**Alberto Velando**

Spain

**Mark Viney**

UK

**Alan Walker**

UK

**Chengshu Wang**

China

**Phurpa Wangchuk**

Australia

**Daniel Wangpraseurt**

Denmark

**Naomi Ward**

USA

**Nicole Wäschke**

Sweden

**Gerald Wilkinson**

USA

**Carrie Woods**

USA

**Hwan Su Yoon**

South Korea

